# Complete mitochondrial genome of red panda (*Ailurus fulgens*) and its phylogenetic analysis

**DOI:** 10.1080/23802359.2019.1629345

**Published:** 2019-07-12

**Authors:** Zuxiang Jin, Huailiang Xu, Diyan Li, Meng Xie, Mingwang Zhang, Qingyong Ni, Yongfang Yao

**Affiliations:** a College of Life Science, Sichuan Agricultural University, Ya’an, China;; b College of Animal Science and Technology, Sichuan Agricultural University, Chengdu, China

**Keywords:** Mitochondrial genome, red panda, phylogenetic analysis

## Abstract

In this study, we reported the complete mitochondrial genome sequence of a red panda (*Ailurus fulgens*). The mitogenome (GenBank accession number MK886830) length is 16,517 bp and exhibits the typical structure of mammalian mitochondrial genomes contains 13 protein-coding genes, 22 transfer RNAs, two ribosomal RNAs, and one control region. The tRNA^Ser (AGY)^ gene failed to form the typical clover-leaf secondary structure as it lacked the dihydrouridine (DHU) arm. phylogenetically, our sequence cluster together with genus *Ailurus*, which showed a closer genetic relationship. The mitogenome provides new data to further elucidation and understand the phylogeny of red pandas.

The red panda (*Ailurus fulgens*) is a carnivorous animal that mainly feeds on bamboo leaves and shoots (Roberts and Gittleman 1984). It primarily distributed in China (Tibet, Yunnan, and Sichuan) while the distribution is very narrow due to fragmentation of habitat and Peculiar feeding. Therefore, in view of its population size, the IUCN classified it as Endangered (EN) Species (Glatston et al. 2015) and listed as category II species in China. Morphological and molecular studies displayed the classification of red pandas have range relationships to the Ursidae, procyonids and mephitids (Decker and Wozencraft 1991; Dragoo and Honeycutt 1997; Delisle and Strobeck 2005). In this study, we sequenced the complete mitogenome of red panda and constructed Phylogenetic tree, which can be used as a reference for the analysis of the red pandas classification.

The muscle tissue of the red panda was collected from a natural death individual in Hailuogou (N29°36′30″, E102°04′7″) of Gongga Mountains, stored in −80 °C refrigerator of Zoology Laboratory of Sichuan Agricultural University, China. We successfully designed 15 specific primer pairs using the mitochondrial genome of *A. ful* (AM711897) and Genomic DNAs were extracted by phenol-chloroform protocol (Sambrook et al. 1990). We manually edited and assembled the DNA sequences using DNAStar software (Burland 1999) and Phylogenetic tree was constructed using Mega 10.0 (Kumar et al. 2018).

The newly generate mitogenome sequence (GenBank accession number MK886830) was 16,517 bp and the base composition is A (33%), T (29.54%), C (24.20%), and G (13.27%). Genes arrangement are similar to other mammals that contain 13 protein-coding genes, 22 transfer RNAs, two ribosomal RNAs, and one control region (Anderson et al. 1981). Ten PCGs were started with the typical ATG codon while *NADH3* and *NADH5* genes initiated with ATT, *NADH2* began with ATA. Meanwhile, all PCGs were terminated with typical TAA or TAG, except for *COIII*, *NADH3*, *NADH4*, which ended with incomplete stop codon T––, and *CYTB* stoped with AGA. In this mitogenome, *NADH6* and eight tRNA genes were encoded on the L-strand, whereas the H-strand encoded two rRNAs, 12 PCGs and 14 tRNAs. All tRNAs were folded into the typical clover-leaf secondary structure, except for the tRNA^Ser (AGY)^ gene as it lacked the dihydrouridine (DHU) arm, which is common phenomenon in other carnivorous (Chen and Zhang 2012; Wu et al. 2018). *12S rRNA* and *16S rRNA* were 966 bp and 1575 bp that were separated by the tRNA^Val^ gene. D-loop was located between tRNA^Pro^ and tRNA^Phe^, with a length of 1071 bp.

Phylogenetic analysis included the complete mitogenome of three red pandas and other nine species from Caniformia, using *Proteles cristata* as outgroup (Figure 1). The neighbour-joining (NJ) analysis exhibited that our sequence cluster together with genus *Ailurus* and then formed a sister relationship with Mephitidae that consistent with the previous reports (Flynn et al. 2005). Furthermore, our study characterized the *Ailurus fulgens styani* mitochondrial genome sequence, which can be used as a reference for the classification of red pandas and contributes to species conservation.

**Figure 1.**
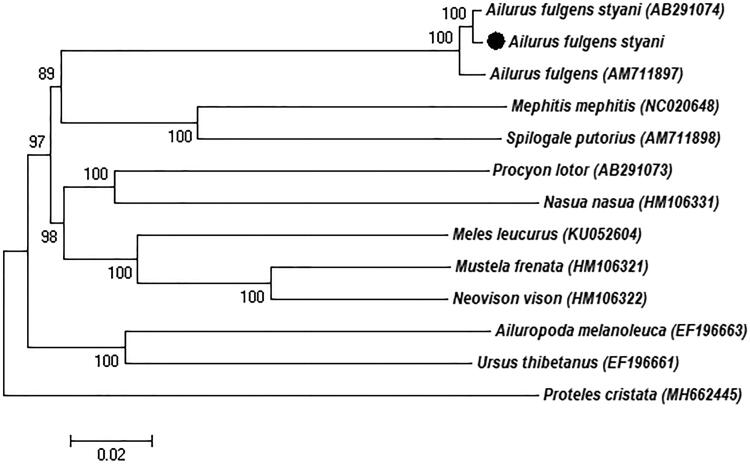
Neighbour-joining (NJ) phylogenetic tree based on the complete mitochondrial genome of the three red panda sequences and other nine Caniformia species sequences. *Proteles cristata* was served as outgroup. Numbers at the branches indicated the bootstrapping values with 1000 replications. GenBank accession numbers were given in the parentheses. Filled circle represented a sequence from this study.
